# Proteins that accumulate with age in human skeletal-muscle aggregates contribute to declines in muscle mass and function in *Caenorhabditis elegans*

**DOI:** 10.18632/aging.101141

**Published:** 2016-12-15

**Authors:** Srinivas Ayyadevara, Meenakshisundaram Balasubramaniam, Pooja Suri, Samuel G. Mackintosh, Alan J. Tackett, Dennis H. Sullivan, Robert J. Shmookler Reis, Richard A. Dennis

**Affiliations:** ^1^ Central Arkansas Veterans Healthcare System, Little Rock, AR 72205, USA; ^2^ Reynolds Institute on Aging, Dept. of Geriatrics, University of Arkansas for Medical Sciences, Little Rock, AR 72205, USA; ^3^ BioInformatics Program, University of Arkansas at Little Rock and University of Arkansas for Medical Sciences, Little Rock, AR 72205, USA; ^4^ Department of Biochemistry & Molecular Biology, University of Arkansas for Medical Sciences, Little Rock, AR 72205, USA; ^5^ Geriatric Research, Education and Clinical Center, Central Arkansas Veterans Healthcare System, Little Rock, AR 72205, USA

**Keywords:** protein aggregation, skeletal muscle, aging, sarcopenia, proteostasis

## Abstract

Protein aggregation increases with age in normal tissues, and with pathology and age in Alzheimer's hippocampus and mouse cardiac muscle. We now ask whether human skeletal muscle accumulates aggregates with age. Detergent-insoluble protein aggregates were isolated from *vastus lateralis* biopsies from 5 young (23–27 years of age) and 5 older (64–80 years) adults. Aggregates, quantified after gel electrophoresis, contain 2.1-fold more protein (*P*<0.0001) when isolated from older subjects relative to young. Of 515 proteins identified by liquid chromatography coupled to tandem mass spectrometry, 56 (11%) were significantly more abundant in older muscle, while 21 (4%) were depleted with age (each *P*<0.05). Orthologs to seven of these proteins were then targeted in *C. elegans* by RNA interference. Six of the seven knockdown treatments decreased protein aggregation (range 6–45%, *P*<0.01 to <0.0001) and increased muscle mass (range 1.5- to 1.85-fold, *P*<0.01 to <0.0001) in aged nematodes, and rescued mobility (range 1.4 to 1.65-fold, *P*≤0.0005 each) in a nematode amyloidopathy model. We conclude that specific aggregate proteins, discovered as differentially abundant in aging human muscle, have orthologs that contribute functionally to aggregation and age-associated muscle loss in nematodes, and thus can be considered potential drug targets for sarcopenia in humans.

## INTRODUCTION

Age-associated muscle loss, or sarcopenia, results in functional decline that increases the risk for falls, disability, and mortality in older adults [1,2]. This problem is clearly influenced by factors such as diet, physical activity, genetics, and comorbid health conditions [3]. However, much less is known about the underlying etiology. Aging has detrimental effects on myofibers, satellite cells, and muscle protein synthesis [4-6]. These effects may be due to dampened levels of growth factors needed for muscle growth and regeneration, or heightened levels of inflammation mediators, which can induce catabolism [7-10].

Several age-associated diseases, particularly those involving neurodegeneration, feature the accumulation of protein aggregates in affected tissues [11]. Interestingly, similar pathology is also seen for inclusion body myositis, an age-associated degenerative skeletal muscle disease, whose protein aggregates contain the amyloid β peptide characteristic of Alzheimer's disease [12]. In diseased neurons and muscle fibers, aggregation is exacerbated by disruption of proteostasis systems responsible for repair or clearance of misfolded and damaged proteins [12-16]. Muscle health is expected to be highly reliant on these processes since it reflects a lifetime of continuous mechanical and metabolic stress [17,18]. However, a causal connection between protein aggregation and muscle aging or sarcopenia has yet to be established.

In the current investigation, we examined protein aggregation that accompanies muscle aging and assessed whether it might contribute to age-associated loss of muscle mass and function. This possibility was suggested by our recent studies which identified and quantified proteins in cardiac muscle aggregates that accrue with aging and hypertension in mice [19]. Our work and that of others has also shown that protein aggregation accumulates with normal aging in the nematode *Caenorhabditis elegans* and in nematode models of protein-aggregation pathologies [16,20,21]. The current study extends our investigation of protein aggregation to human muscle, with three objectives: 1) determine if aging is associated with increased protein aggregation in human skeletal muscle; 2) identify muscle-aggregate proteins that are differentially abundant with age; and 3) identify nematode orthologs of selected human aggregate proteins, and test their mechanistic involvement in protein aggregation and age-associated loss of muscle mass and function in *C. elegans*.

## RESULTS

### Protein aggregation increases with age in human muscle

**Figure 1:**
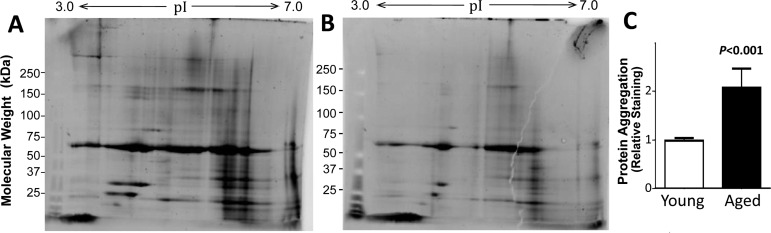
The protein content of muscle aggregates from older subjects was higher than that from young adults Sarcosyl-insoluble aggregates were isolated from muscle of old (**A**) and young (**B**) donors (pooled, *N*=2 per group), dissolved in ampholyte buffer, resolved on 2D gels, and stained for total protein. Aggregates from 5 individuals per group were resuspended and heated to 95°C in Laemmli buffer (containing 2% SDS, w/v, and 0.5% Δ-mercaptoethanol, v/v), and resolved on 1D gels to quantify total aggregate protein (**C**). Mean ±SD is shown after normalization to mean total aggregate protein for the young group.

Protein aggregation was compared for muscle from young and old adults after isolation of aggregates that were insoluble in a strong ionic detergent (1% sarcosyl) from equal wet weights of initial muscle protein. Aggregates from two young (23 and 26 year-old males) and two old adults (a female age 65, and a male age 68) were pooled and resolved by 2D gel electrophoresis. Staining the gels for total protein showed a greater diversity of proteins, and higher abundance for most of these, in muscle aggregates from aged adults than in aggregates from young adults (Figure 1, A and B). Total muscle-aggregate protein was then quantitated for individuals, comparing samples from 5 young adults (ages 23–27 years; one female) and 5 older adults (ages 64–80 years, two females) on 1-D gels. The results confirmed that protein aggregation was significantly higher (2.1±0.07 fold, *P*<0.0001) for the older group than for young adults (Figure 1C).

### Specific proteins are enriched with age in aggregates from human muscle

Aggregate proteins were identified, and their relative abundances estimated, by mass spectrometry of young and old human muscle samples pooled by age group (as described above). Muscle-aggregate proteins were dissolved in strong detergent and reducing agent, electrophoresed in one dimension, and gel slices were digested *in situ* with trypsin. Peptides were then identified by matching fragmentation patterns to a reference database within Mascot software (Matrix Science, Boston MA). Spectral counts (the number of peptide identifications per protein) allow an unbiased comparison of relative abundances for any given protein, between young and old human muscle. A total of 515 proteins were positively identified from muscle aggregates. When results were filtered to require ≥1.5-fold difference by age and ≥3 peptide identifications for at least one group, 168 proteins were more abundant in aggregates from old muscle than young (mean old/young ratio ± SEM = 7.0 ± 2.4), whereas 60 proteins were reduced in old muscle (old/young ratio = 0.092 ± 0.025).

For each protein, we assessed the significance of spectral-count enrichment at either age (by Chi-squared tests, relative to total aggregate protein); 56 of the 515 proteins (11%) were significantly enriched in muscle aggregates from old donors while 21 (4%) were depleted with age (each *P*<0.05). The complete list of identified proteins is provided in Supplementary Table S1. A partial list of aggregate proteins (Table 1) includes 34 proteins that differ significantly in abundance between young and old muscle. Half of these (17) are cytoskeletal and/or involved in muscle contraction, and another (LMCD1) regulates muscle differentiation. Eight are involved in protein homeostasis: 5 heat-shock protein isoforms, peroxi-redoxin, and 2 ubiquitin-conjugating enzymes.

**Table 1:** Protein aggregates from muscle of young and old adults and their *C. elegans* orthologs The last 3 columns reflect spectral counts from LC-MS^2^ analysis of sarcosyl-insoluble aggregates. Bold font indicates proteins for which *C. elegans* orthologs were targeted by RNAi to assess effects on protein aggregation and muscle mass.

Protein	Function	Nematode Orthologs	LC-MS/MS Spectral Counts		
			Young Muscle	Old Muscle	*Chi^2^ (≈ FE_2_) *P<*
14-3-3 Proteins, isoforms ε, γ	Bind >200 signaling prot's	**FTT-2**, PAR-5	3	19	0.001
Adenylosuccinate synthetase isozyme 1	Purine biosynthesis	**ADSS-1**	2	17	0.001
Alpha actinin 3	Muscle contraction, fast	―	156	67	0.0001
Ankyrin-2	Cytoskeletal anchoring	**UNC-44**	5	8	NS
Annexin A2	Cytoskeleton, endosomes	―	1	12	0.003
Calsequestrin-2	Muscle contraction, slow	―	1	11	0.004
Carbonic anhydrase 1	Respiration, pH balance	**CAH-1**	7	14	NS
Cofilin-1, cofilin-2	Actin binding/reorganization	―	25	53	0.005
DJ-1 protein, PARK7	Inhibits α-synuclein aggreg.	―	14	30	0.03
Dystrophin, DMD	Muscle cytoskeleton	―	16	5	0.02
Fatty acid-binding protein, heart (FABPH)	Long-chain FA transport	―	17	34	0.03
F-actin capping protein Zβ	Cytoskeletal organization	**CAP-2**	6	14	NS
Filamin C	Actin binding	**FLN-1**	400	258	0.0005
Four and a half LIM domains protein 1	Defect causes myopathies	―	110	143	0.04
Galectin-1	Cell-cell, cell-matrix interact's	―	16	33	0.03
Heat shock proteins beta-2, -3, -6	Muscle-specific small HSPs	―	23	49	0.003
Heat Shock Protein HSP90-α,-β	Protein-refolding chaperones	DAF-21	33	52	0.04
Histones H3.3, H2A1	Chromatin structure	―	17	53	6E–5
L-lactate dehydrogenase B	Cell energy metabolism	―	8	28	0.001
Lamin A/C (LMNA)	Cytoskeletal protein	IFB-1	44	19	0.005
Laminin beta-2	Extracellular matrix	―	30	12	0.01
LIM & cysteine-rich domains prot. 1, LMCD1	Muscle differentiation	**MLP-1**	12	29	0.02
Myosin light chain 5	Muscle contraction	―	10	0	0.002
Myosin light polypeptide 6	Muscle contraction	―	0	27	5E–8
Myozenin-2	Muscle contraction, slow	―	1	21	1E–5
Peroxyredoxin-2	Antioxidant/redox protein	―	12	26	0.04
Phosphatidylethanolamine-binding prot.1, PEBP1	Binds PE>PI ≈ PC	―	15	41	0.001
Troponin T, slow (TNNT1)	Muscle contraction, slow	―	38	125	4E–9
Troponin I, slow (TNNI1)	Muscle contraction, slow	―	65	157	4E–7
Ubiquitin-conjugating enzymes E2-1, E2-2	Protein degradation	**UEV-1**	1	9	0.02
Xylulose reductase	Detoxification, metabolism	**DHS-21**	2	6	NS

* *P* values are Chi^2^ *P* values. For these comparisons, with large and nearly equal spectral-count totals in young (21,940) and old (21,957) LC-MS/MS analyses, Chi^2^ *P* was nearly equal to 2-tailed Fisher Exact test (FE_2_) *P*.

### Candidate-gene knockdowns in C. elegans relieve age-associated aggregation

We selected nine *C. elegans* proteins orthologous to proteins identified in human muscle aggregates (Table 1, candidates listed in bold), for which the corresponding nematode genes are available in the Ahringer RNA-interference (RNAi) library [58]. These comprise four aggregate proteins that were significantly more abundant in old muscle (FTT-2, ADSS-1, MLP-1, and UEV-1), four that showed a suggestive enrichment with age (UNC-44, CAH-1, CAP-2, and DHS-21), and one that was more abundant in young muscle (FLN-1). Gene expression was individually knocked down in nematodes by life-long RNAi, beginning with hatching of L1 larvae from eggs, in strain AM141. This strain expresses Q40::YFP (a tract of 40 glutamines fused in-frame to yellow fluorescent protein) in body wall muscle and is considered a model of Huntington-like protein aggregation as it accumulates fluorescent aggregates during days 1–5 of adult life.

**Figure 2:**
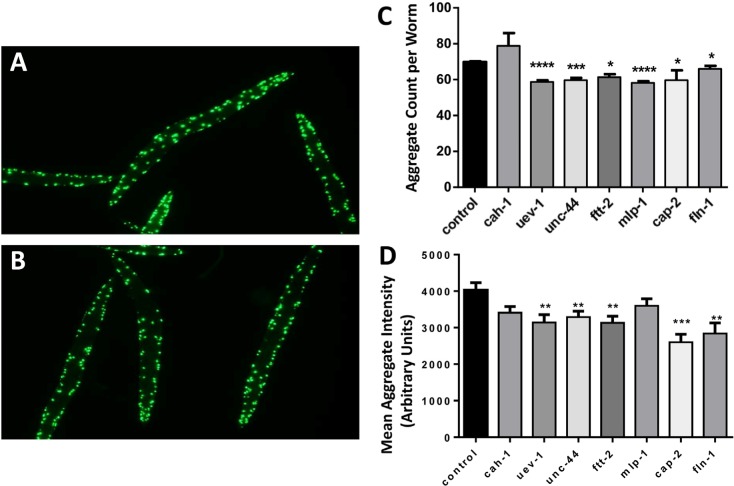
Nematodes exposed to RNAi targeting human muscle-aggregate orthologs have fewer and smaller protein aggregates Fluorescent aggregates (Q40::YFP foci) are shown in strain AM141 (*N*=40–65 worms per group) after maintenance from hatch on bacteria carrying either control (empty vector) (**A**) or *uev-1* RNAi knockdown (**B**) expression plasmid. Histograms show the mean ± SEM of the number of aggregate foci (**C**) and fluorescent intensity per aggregate (**D**). Significance of differences from control: *P<0.05; **P<0.02; ***P<0.001; ****P<0.0001.

Two knockdowns (targeting *adss-1* and *dhs-21*) delayed development and were not pursued. Six of the remaining seven gene knockdowns significantly reduced protein aggregation, as illustrated in Figure 2. Representative images are shown for day-3 adult controls (panel A), in which aggregates are consistently a bit larger and more numerous than in worms knocked down for a candidate gene (*uev-1*) (panel B). Computer quantitation of images indicated significant reductions in the number of aggregates (by 6–17%; *P-*values of 0.03 to <0.0001; Figure 2C) for all RNAi targets except *cah-1*, accompanied by reduced intensity of Q40::YFP fluorescence per aggregate (by 11–45%; *P-*values 0.01 to <0.001; Figure 2D) for all targets except *cah-1* and *mlp-1*.

### Gene knockdowns in C. elegans which decrease protein aggregation prevent age-associated loss of muscle mass

**Figure 3:**
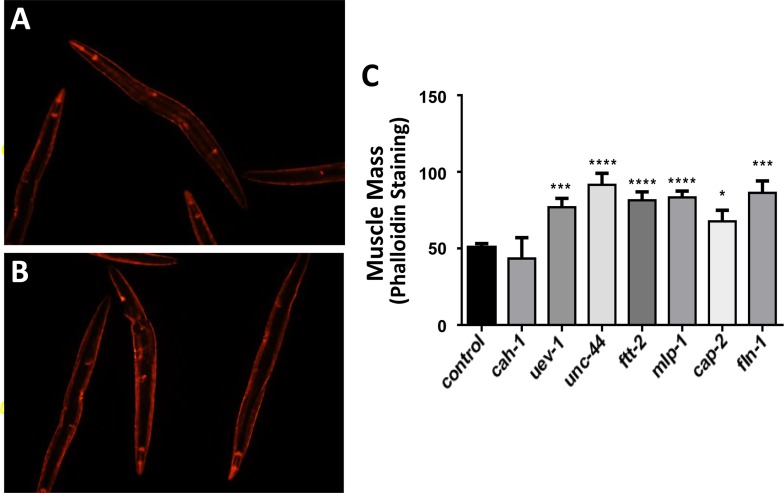
Nematodes exposed to RNAi targeting human muscle-aggregate orthologs have increased muscle mass Muscle was stained with rhodamine-tagged phalloidin in wild-type worms (strain N2, *N*=25 – 35 per group) as illustrated for control (**A**) and RNAi-exposed worms (targeting *uev-1* in panel **B**). Staining intensity (mean ± SEM) of muscle mass is presented (**C**). Significance of differences from control: *P<0.05; **P<0.02; ***P<0.001; ****P<0.0001.

Targeting the same seven genes with RNAi also preserved muscle mass and function in aged nematodes (Figure 3), which otherwise lose 25–30% of muscle mass by day 3, and >70% by day 9 of adult life. Wild-type nematodes (strain Bristol N2) were exposed from hatch to empty vector (panel A) or gene-specific RNAi constructs (e.g. *uev-1*, panel B) and stained with rhodamine-tagged phalloidin to quantify muscle at 3 days of adult age. Knockdown of six genes (again, all except *cah-1*) consistently and significantly increased muscle mass relative to controls by 50–85% (*P* values ranged from 0.01 to <0.0001; panel C). Essentially the same preservation in muscle quantity was observed, without staining, in transgenic reporter strain RW1596 that expresses green fluorescent protein (GFP) driven by the muscle-specific *myo-3* promoter (data not shown).

### Gene knockdowns in C. elegans which decrease protein aggregation also counteract age-associated loss of muscle function

**Figure 4:**
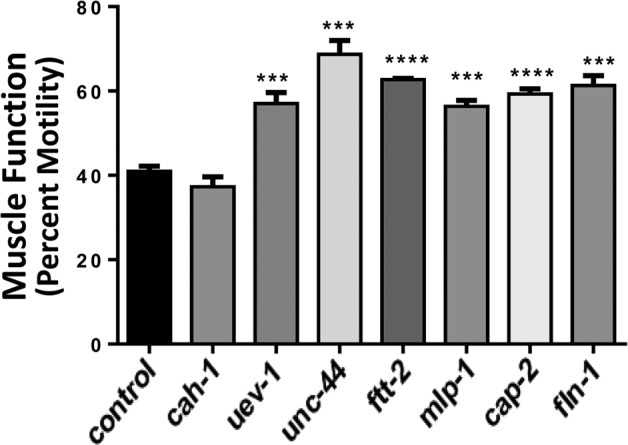
Nematodes exposed to RNAi targeting human muscle-aggregate orthologs are resistant to amyloidopathy-induced paralysis Muscle-specific induc-tion of human Aβ_1-42_ expression during development causes paralysis to ensue over the ensuing 29 – 48 hr. Bars show the extent (mean ± SEM) to which treated worms were protected from amyloid-mediated paralysis, which left only 41% of control worms motile (*N* = 40 – 60 per group). Significant differences from control: ***P = 0.0005; ****P<0.0001.

Finally, muscle function was assessed in nematode strain CL4176, which becomes paralyzed due to the formation of amyloid aggregates ∼48 hrs after induced expression of human Aβ_1–42_ peptide in muscle. Knockdown from hatch, of the same six genes that significantly decreased protein aggregate counts (Figure 2) and increased muscle mass (Figure 3) also consistently rescued worms from amyloid paralysis, resulting in 39–67% (average of 47%) more motile worms per group at 48 hr post-induction (each *P*≤0.0005) than were seen among controls (Figure 4). By the time control worms reached zero motility (100% paralysis, 48 hr post-induction), 5–20% of RNAi-exposed worms remained motile (data not shown).

## DISCUSSION

We assessed protein aggregation in human skeletal muscle from young-adult and elderly subjects, and found more than twice as much aggregate (as a fraction of total muscle protein) in the older samples. Among proteins identified in aggregates at each age, 15% differed with nominal significance (α <0.05) between the age groups, far more than the 5% expected by chance. We identified conserved nematode orthologs of age-differential human proteins and assessed their functional roles by RNA interference.

Previous proteomic comparisons of young and aged muscle for rodents and humans found 3 – 23% of soluble proteins altered in abundance with age [22-26]. In the present analysis, 43% of the 515 proteins identified in sarcosyl-insoluble muscle aggregates differed by at least a 1.5-fold in abundance between age groups, and 15% were significantly different (α <0.05). These results suggest that insoluble protein aggregates may be particularly susceptible to the effects of aging and could play a role in sarcopenia analogous to their role in the pathology of neurodegenerative diseases [27,28]. This possibility was directly supported by disruptions of gene expression for *C. elegans* orthologs of human aggregate proteins: six of the seven tested knockdowns reduced protein aggregation and improved muscle-mass retention and resistance to amyloid-induced paralysis in aged nematodes.

Misfolded and damaged proteins are normally cleared by the ubiquitin-proteasome system (UPS), or failing that, by autophagy [29]. However, age-associated declines have been found in autophagy [30,31], lysosomal function [32], and proteasomal activity [33], resulting in deficient muscle growth and accumulation of insoluble polyubiquitinylated protein aggregates [34]. If autophagy is deficient, protein aggregation, abnormal mitochondria and oxidative stress all become elevated in mouse muscle [35,36]. We found several proteins involved in UPS, autophagy, and protein homeostasis to be increased in aggregates from aged skeletal muscle, relative to those from young muscle, suggesting dysfunctional protein-clearance machinery (Table 1 and Supplementary Table S1). Proteins enriched in aged muscle aggregates include proteasomal subunits; E1 ubiquitin modifier enzyme and E2 ubiquitin ligase; Cu/Zn- and Mn-superoxide dismutases (SODs); and small heat shock proteins (HSPs) including 10-kD HSP and HSPs β2, β3, and β6. The small HSPs work as chaperones in maintaining protein homeostasis, and are implicated in the development and maintenance of muscle mass [37-40]. We observed similar HSP enrichments in mouse cardiac-muscle aggregates with age [19].

Several proteins that we tested in nematodes (carbonic anhydrase, ankyrin-2, filamin, and f-actin capping protein) may be structurally prone to aggregation and might negatively impact the health of muscle and other tissues by increasing the total aggregate burden. Carbonic anhydrase was the only protein tested whose effects were insignificant in the nematode models. However, its presence in muscle aggregates may reflect formation of amyloid fibrils, as were noted previously for its bovine ortholog [41]. Similarly, ankyrin-2 may be structurally disposed to aggregate since ankyrin-like domains can target proteins to aggrosomes in human cells [42].

Filamin and f-capping protein may have been recruited to protein aggregates through their numerous interactions with actin and other cytoskeletal proteins. Cytoskeletal proteins are a major component of aggregates in human myofibrillar myopathies [43], and were the most numerous functional category we observed here (Table 1). Furthermore, mutation of filamin can cause myopathy through toxic protein aggregation [44]. F-actin capping protein Zβ has not, to our knowledge, been previously implicated in aggregation or human myopathies, although its expression is elevated in early-stage Alzheimer's disease [45].

Proteins with known roles in proteostasis (HSPs, ubiquitin conjugating enzyme, 14-3-3 proteins, and LIM and cysteine-rich domains protein) could occur in aggregates due to their involvement in aggregate removal by proteasomes or autophagy [46,47]. Ubiquitin conjugating enzymes perform one of the steps necessary for ubiquitin addition which targets proteins to the proteasome for degradation. In nematodes, RNAi against conjugating enzymes slowed the growth of protein aggregates [48]; whereas in mice, defects in the ubiquitin system caused accumulation of myofibrillar proteins and myopathy [49]. 14-3-3 proteins may also help to direct misfolded proteins to clearance pathways [50]; these proteins are present in aggregates associated with myopathy and multiple neurodegenerative diseases [43;51].

Misfolded proteins, including aggregates, may be cleared by autophagosomes if not degraded by proteasomes. LIM and cysteine-rich domains proteins may participate in this process since disrupting their expression blocks autophagy in mouse muscle cells [52]. Interestingly, disrupting *FHL1* expression blocks muscle cell differentiation, and its mutation results in myopathy associated with protein aggregation [53,54]. Aging impairs the ability of cells to deal with misfolded proteins [55], largely due to impairment in the ubiquitin-proteasome system and autophagy.

By comparing aggregate amounts and compositions across human aging, and assessing functional impacts of aggregate-associated proteins through nematode studies, we were able to demonstrate that age-dependent accumulation of aggregates in muscle can underlie the loss of muscle mass and function that are commonly observed to accompany human aging. Skeletal muscle mass is expected to be influenced by relative rates of protein synthesis and degradation but the current study provides the first evidence that specific proteins are involved in the formation of insoluble protein aggregates that are toxic to muscle. Multiple proteins of diverse function were functionally implicated in protein aggregation, suggesting that the key causal parameter is the aggregate burden itself, rather than an upstream regulator of aggregation. Furthermore, since dampen-ing production of aggregate proteins produced marked improvements in muscle mass and function, we propose that protein aggregation may provide attractive targets for therapeutic intervention in age-dependent sarco-penia. We conclude that protein aggregation is not unique to neurodegenerative disease and genetic myopathies, but is also characteristic of normal muscle aging and may contribute to muscle loss and functional decline with age.

## METHODS

### Human subjects

This study was conducted in accordance with the ethical standards of the Declaration of Helsinki and national and international guidelines. After provision of informed consent, human muscle samples were collected under a protocol approved by the Institutional Review Board (IRB) of the University of Arkansas for Medical Sciences. Samples were de-identified for analysis at the Central Arkansas Veterans Healthcare System, and the CAVHS IRB determined the ensuing proteomic studies to be non-human research.

All subjects were medically cleared for participation by a study physician. Eligible individuals were between 19 – 30 years of age (“young” group) or 60 – 80 years of age (“old” group), had body mass indices of <30 kg/m^2^, and were non-smokers. Individuals were excluded for history of lidocaine allergy or bleeding disorder, or for taking anti-inflammatory or anti-coagulant medications within seven days of their appointment. Subjects were required to abstain from strenuous physical activity for 3 days and alcohol consumption for 2 days prior to undergoing muscle biopsy. Muscle was sampled from the *vastus lateralis* under local anesthesia (1% lidocaine-HCl, buffered to pH 9:1 with sodium bicarbonate) using a 6-mm Bergstrom trocar (Millennium Surgical, Narberth, PA) with mild suction. The tissue was immediately stabilized in RNAlater (Life Technologies, Grand Island, NY), held overnight at 4°C, and then stored at −20°C until processing.

### Human aggregate purification, protein separation and quantitation

Muscle (15 mg per specimen) was pulverized in a dry-ice cooled mortar and suspended in cold stabilizing buffer (20-mM Hepes pH 7.4, 0.3-M NaCl, 2-mM MgCl_2_, 1% NP40 (w/v)) containing phosphatase/prote-ase inhibitors (CalBiochem). Crushed tissue was lysed on ice using a Teflon homogenizer (2 × 10 sec) and sonication (3 × 10 sec). After centrifugation (5 min, 2000*g*) to remove debris and organelles, protein concentrations of supernatants were determined (Bradford Protein Assay, Bio-Rad). Aggregate proteins were collected from equal amounts of total protein by centrifugation (15 min, 14000g), and pellets were suspended in ionic detergent buffer (1% sarcosyl (v/v); 0.1-M Hepes, pH 7.4; 5-mM EDTA) with protease inhibitors. Detergent-insoluble aggregate proteins were then pelleted by ultracentrifugation (30 min, 100,000 x *g*) and resuspended in 125-μl ampholyte buffer (8M urea; 2% CHAPS; 40-mM DTT; 0.2% w/v Biolyte, pH range 3–10). Proteins were separated by isoelectric focusing (pH 3–10) and then by orthogonal gradient gel electrophoresis (1% sodium dodecyl sulfate (SDS), 4–12% polyacrylamide). The separated proteins were visualized after staining with SYPRO Ruby (Invitrogen) and fluorescence intensities were quantified using ImageJ software (http://imagej.nih.gov).

### Identification of proteins in human muscle aggregates

Individual proteins in the aggregate fractions were resolved and identified as follows. Sarcosyl-insoluble aggregate proteins (the 100,000 x *g* pellet described above) were dissolved by heating (5 min, 95°C) in Laemmli buffer containing 2% SDS (w/v) and 0.5% Δ-mercaptoethanol (v/v). Proteins were resolved on gradient gels (1% SDS, 4‒12% polyacrylamide) and stained with SYPRO Ruby or Coomassie Blue. Each lane was robotically partitioned into 1-mm slices and the proteins were digested *in situ* with trypsin [16]. The resulting peptides were analyzed by high-resolution LC tandem MS (ThermoFisher Orbitrap Fusion Tribrid) as reported previously [56;57]. Proteins were identified by MASCOT (www.matrixscience.com) matching of pep-tide fragments to a database of predicted patterns [57].

### Nematode strains

The wild-type *C. elegans* and two disease-model strains used to assess the role of protein aggregation in muscle aging were obtained from the Caenorhabditis Genetics Center which is funded by the NIH Office of Research Infrastructure Programs (P40 OD010440). Strains were maintained on 2% agar plates containing nematode growth medium overlaid with *E. coli* (strain OP50) at 20°C. Cohort age was synchronized by alkaline-hypochlorite lysis of well-fed gravid hermaphrodites at day 3 post-hatch (day 1 of adulthood) to release unlaid eggs. Eggs were plated on 100-mm plates seeded with empty-vector control or RNAi bacteria (Ahringer RNAi library [58]) designed to knock down production of each targeted protein. Nematode images were captured using an Olympus BX51 fluorescence microscope equipped with an Olympus DP71 camera, and aggregates were quantified using ImageJ software (http://imagej.nih.gov).

Nematode strains were selected to facilitate measurement of the effects of aging on muscle protein aggregation, muscle mass, and paralysis. Wild-type strain Bristol-N2 (N2) is a relatively long-lived DRM stock of Bristol-N2 maintained by the Caenorhabditis Genetics Center. Strain AM141 (*unc-54p*/Q40::YFP) expresses a tract of 40 glutamines (Q40) fused in-frame to yellow fluorescent protein (YFP), constitutively in muscle. This strain emulates the threshold pathologic mutation of huntingtin protein that elicits Huntington's disease, and forms yellow fluorescent aggregates in muscle progressively with adult age [16]. Aggregate foci were counted, either manually or using DotCount (http://reuter.mit.edu/software/dotcount/) software. Strain CL4176 (*smg-1*^ts^ [*myo-3p*/Aβ_1–42_/long 3′-untranslated region] ) expresses human amyloid β_1–42_ (Alzheimer's disease peptide, Aβ_1–42_) in muscle after induction by temperature upshift from 20° to 25°C at the L3/L4 transition [16]. Induction of Aβ_1–42_ expression causes paralysis that was measured at 29 h post-induction, as movement of the head but not the body in response to touch. All experiments were performed at least in triplicate.

### Statistical analysis

Differences between groups were assessed for significance by the Fisher-Behrens heteroscedastic *t* test (appropriate to samples of unequal or unknown variance). Differences in peptide abundance, based on mass spectrometry spectral counts, were assessed for significance by chi-squared or 2-tailed Fisher exact tests. Significance values reported are nominal *P* values without adjustment for multiple comparisons. To calculate mean fold differences of proteins that were uniquely present in one age group, 0.1 was substituted for zero to prevent division by zero.

## SUPPLEMENTARY METHODS AND FIGURES




