# Supplementary material for: Proteins that accumulate with age in human skeletal-muscle aggregates contribute to declines in muscle mass and function in Caenorhabditis elegans
Source: Aging (Albany NY). 2016 Dec 15;8(12):3486–96. doi: 10.18632/aging.101141 (PMC5270681; doi:10.18632/aging.101141)
Supplement: Supplementary file 1 [file aging-08-3486-s001.pdf]

SUPPLEMENTARY MATERIAL

Please follow the link in Full text version to see the data of Supplementary Table of this manuscript.
